# Higher Body Mass Index is associated with increased arterial stiffness prior to target organ damage: a cross-sectional cohort study

**DOI:** 10.1186/s12872-023-03503-5

**Published:** 2023-09-14

**Authors:** Nejc Piko, Sebastjan Bevc, Radovan Hojs, Tadej Petreski, Robert Ekart

**Affiliations:** 1 Department of Dialysis, Clinic for Internal Medicine, University Medical Centre Maribor, Ljubljanska Ulica 5, 2000 Maribor, Slovenia; 2 Department of Nephrology, Clinic for Internal Medicine, University Medical Centre Maribor, Ljubljanska Ulica 5, 2000 Maribor, Slovenia; 3 Medical Faculty, University of Maribor, Taborska Ulica 8, 2000 Maribor, Slovenia

**Keywords:** Obesity, Atherosclerosis, Arterial stiffness, Cardiovascular disease, Chronic kidney disease

## Abstract

**Background:**

Obesity is associated with several neurohumoral changes that play an essential role in organ damage. Increased arterial stiffness causes functional vessel wall changes and can therefore lead to accelerated target organ damage as well. Whether obesity causes an independent increase in central arterial stiffness is, however, not yet fully known.

**Methods:**

One hundred thirty-three patients (63.2% male) were included. Body Mass Index (BMI) was defined as body weight in kilograms, divided by the square of body height in meters. Chronic Kidney Disease Epidemiology Collaboration creatinine 2009 equation was used to estimate the glomerular filtration rate (eGFR). Non-invasive applanation tonometry was used for arterial stiffness measurements (Sphygmocor Atcor Medical, Sydney, Australia). All patients underwent coronarography.

**Results:**

The mean age of our patients was 65.0 ± 9.2 years. Their mean BMI was 28.5 ± 4.4 kg/m^2^, eGFR 75.5 ± 17.2 ml/min/1.73 m^2^ and ankle-brachial index (ABI) 1.0 ± 0.1. Their arterial stiffness measurements showed mean carotid-femoral pulse wave velocity (cfPWV) 10.3 ± 2.7 m/s, subendocardial viability ratio (SEVR) 164.4 ± 35.0%, and pulse pressure (PP) 47.8 ± 14.5 mmHg. Spearman's correlation test revealed a statistically significant correlation between BMI and SEVR (*r* = -0.193; *p* = 0.026), BMI and cfPWV (*r* = 0.417; *p* < 0.001) and between BMI and PP (*r* = 0.227; *p* = 0.009). Multiple regression analysis confirmed an independent connection between BMI and cfPWV (B = 0.303; *p* < 0.001) and between BMI and SEVR (B = -0.186; *p* = 0.040). There was no association between BMI and kidney function, ABI, or coronary artery disease.

**Conclusion:**

Increased BMI is independently associated with augmented central arterial stiffness and reduced subendocardial perfusion but not with coronary artery disease, kidney function, or ABI.

## Background

Obesity is a complex and multifactorial disease, affecting nearly one-third of the world’s population. The epidemiologic burden of obesity is increasing and it is postulated that by 2030, almost 40% of all adults will be either overweight or obese [1].

Body Mass Index (BMI) values between 25 and 30 kg/m^2^ indicate that a person is overweight. World Health Organization (WHO) defines obesity as excessive fat accumulation that can impair health and is diagnosed at BMI ≥ 30 kg/m^2^ [2, 3]. A major drawback of BMI is that it does not account for body composition. It can therefore be elevated in patients with high muscle-low body fat content and vice versa; it can be low in patients with low muscle-high body fat content, frequently labelled as sarcopenic obesity. Nonetheless, BMI has several advantages, including low cost, affordability, and reproducibility, making it the universal and most commonly used marker of obesity [4].

Obesity substantially increases the risk of metabolic diseases (type 2 diabetes mellitus and fatty liver disease), cardiovascular diseases (arterial hypertension, ischemic heart disease, stroke), musculoskeletal disease (osteoarthritis), Alzheimer dementia, chronic kidney disease (CKD), obstructive sleep apnea, and several malignancies (breast, ovarian, prostate, liver, kidney and colon) [5]. Besides increased healthcare costs, obesity is associated with unemployment, numerous social disadvantages, and reduced quality of life [6]. Importantly, several organizations (for example, World Obesity Federation, American and Canadian Medial Associations) have declared obesity an independent, chronic progressive disease that is more than just a risk factor for other diseases [7]. Prompt intervention is, therefore, necessary to reduce the negative effects of obesity on the health of the global population [5].

Arterial stiffness develops from an intricate interaction between structural and functional vessel wall alterations, which are inherently linked to the process of atherosclerosis [8]. Vascular wall inflammation and oxidative stress, increased collagen production and deposition, decreased elastin synthesis and proliferation of vascular smooth muscle cells are all pivotal steps in structural changes of the arterial vascular tree [8, 9]. These vascular alterations are driven by hemodynamic factors, such as arterial hypertension and are amplified through the presence of common diseases, such as diabetes, or simply aging itself [10]. Decreased compliance of central vasculature alters arterial pressure and flow dynamics and impacts cardiac performance and coronary perfusion [11]. Additionally, increased pulsatility can cause accelerated, mechanically-induced target organ damage, independent of the atherosclerosis [12]. Arterial stiffening is therefore a marker of increased cardiovascular risk and is connected to myocardial infarction, heart failure, kidney impairment, stroke, dementia, and, ultimately, higher mortality [13–15].

Obesity is an important predictor of atherosclerosis [16]. Besides well-known association with traditional atherosclerosis risk factors, obesity can lead to various neurohumoral changes (such as hyperinsulinemia, hyperglycemia, increased activation of renin–angiotensin–aldosterone and sympathetic systems, maladaptive immune and imflammatory responses and increased oxidative stress) [17]. These changes could be a major driving force behind increased arterial stiffness in obese patients [18].

## Methods

### Aim of the study

The aim of the study was to assess whether increased BMI can lead to increased arterial stiffness and if this connection is independent of traditional atherosclerosis risk factors. Additionally, we wanted to determine if there was any connection between BMI and target organ damage, such as coronary artery disease (defined by coronarography), peripheral arterial perfusion (determined by ankle-brachial index – ABI), and kidney function (determined by estimated glomerular filtration rate (eGFR)).

### Study population

In our cross-sectional cohort study, 133 patients were included. All patients were hospitalised at the Department of Cardiology and Angiology at the Clinic for Internal Medicine, University Medical Centre Maribor between March 1^st^, 2016, and February 1^st^, 2020, due to planned elective coronarography (previously positive either cycle ergometry testing or perfusion myocardial scintigraphy).

Exclusion criteria for the study were pregnancy, active malignancy at the time of the study, and age under 18 years. Both atrial fibrillation and aortic stenosis can impact peripheral pulse wave readings and arterial stiffness measurements; patients with these two pathological entities were therefore also excluded from the study [19].

The patient’s medical history along with comorbidities and prescribed medications at the time of inclusion in the study was recorded.

BMI was calculated as the ratio between body weight in kilograms and height in squared meters and was expressed in kg/m^2^.

Before coronarography, patients had their peripheral blood drawn. Analysed laboratory values included serum haemoglobin (g/L), serum lipid profile (total cholesterol, low-density lipoprotein (LDL), high-density lipoprotein (HDL) and triglycerides, all values were expressed in mmol/L), creatinine (umol/L), cystatin C (mg/L), and N-terminal pro-hormone beta natriuretic peptide (NT-proBNP, described in ng/L).

Ankle-brachial index (ABI) was measured by using a previously validated, automated non-invasive waveform analysis device (MESI®, Slovenia) [20]. Blood pressure was measured simultaneously on both calves and the brachial part of the right arm. ABI was calculated automatically as the ratio between the ankle systolic blood pressure and brachial systolic blood pressure. The average value between the left and right leg was then used for statistical analysis.

The glomerular filtration rate was estimated (eGFR) by using the Chronic Kidney Disease Epidemiology Collaboration (CKD-EPI) 2009 creatinine equation.

All patients signed a written informed consent prior to inclusion in the study.

The National Ethics Committee approved the study (N°0120–32/2017/4). The study adhered to the Declaration of Helsinki and was performed per Good Clinical Practice standards.

### Arterial stiffness measurements

Arterial stiffness was measured at the Department of Dialysis, Clinic for Internal Medicine, University Medical Centre Maribor. Non-invasive applanation tonometry was employed to obtain these measurements (Sphygmocor Atcor Medical, Sydney, Australia). All the measurements were performed by two examiners (both medical doctors), between 10 and 12 AM, from Monday to Friday, before coronarography.

Each patient waited 5–10 min in a supine position before the measurements and all electronic gadgets and mobile phones were turned off during the study to prevent distortions in the obtained data. All patients abstained from coffee, cigarettes, heavy meals, and exercise at least 12 h before.

Pulse wave analysis (PWA) was performed on the radial artery. Pulse pressure (PP) was defined as the difference between systolic and diastolic blood pressure. Augmentation pressure (AP) was the difference between the first and second systolic peaks. The augmentation index (AIx) was calculated as the ratio between PP and AP and it was also normalised to a heart rate of 75 beats per minute (AIx@75). The ejection duration (ED) was defined as the duration of the left ventricle systolic ejection. Subendocardial viability ratio (SEVR) was obtained from the ratio between the diastolic and systolic time index [21].

All the measurements had to fulfil quality criteria to be included. Quality indices were: operator index ≥ 80%, average pulse height ≥ 80%, pulse height variation ≤ 5%, and diastolic variation ≤ 5%.

Carotid femoral pulse-wave velocity (cfPWV) was defined as the pulse wave distance between the carotid and femoral artery, divided by pulse transit time (measured electrocardiographically). A subtracted carotid-femoral distance was used ((sternal-femoral)—(carotid-sternal)) [22]. Each patient had three optimal cfPWV measurements, defined by the standard deviation (SD) < 10%. The average cfPWV value was then taken as the study parameter.

### Statistical analysis

For statistical analysis, Statistical Package for Social Sciences version 28.0 was used (SPSS Inc, Chicago, IL, USA). Basic descriptive statistics were used for continuous variables (mean ± SD). Categorical variables were expressed with frequencies and percentages.

The distribution of variables was tested with the Shapiro–Wilk test. Due to the non-normal distribution of our variables, non-parametric tests were used, such as Spearman's correlation test and one-way analysis of variance (ANOVA).

Multiple regression analysis was performed with SEVR, cfPWV, and PP as dependent variables, and BMI, systolic blood pressure, diabetes, age, hyperlipidemia, and eGFR as independent variables. Additionally, BMI groups were created by using quartiles, and the differences between BMI groups were tested by using ANOVA and Chi-squared test. For all tests, a *p*-value of < 0.05 was defined as statistically significant.

## Results

84 patients (63.2%) were male. The most common comorbidities were arterial hypertension (*n* = 105, 78.9%), hyperlipidemia (*n* = 74, 55.6%), diabetes mellitus type 2 (*n* = 29, 21.9%), heart failure (*n* = 15, 11.3%), CKD (*n* = 10, 7.5%) and peripheral arterial disease (*n* = 8, 6.0%).

Most commonly prescribed medications were acetylsalicylic acid (*n* = 115, 86.5%), statins (*n* = 100, 75.2%), beta-blockers (*n* = 93, 69.9%), angiotensin convertase inhibitors (*n* = 77, 57.9%), diuretics (*n* = 49, 36.8%), calcium channel blockers (*n* = 33, 24.8%), angiotensin II receptor blockers (*n* = 23, 17.3%) and alfa channel blockers (*n* = 20, 15.0%). Seven patients (5.3%) were receiving aldosterone antagonists. The most commonly prescribed peroral antidiabetic therapy was metformin (*n* = 22, 16.5%), ten patients (7.5%) had been prescribed insulin.

The mean age of our patients was 65.0 ± 9.2 years (range 27–82), the mean BMI was 28.5 ± 4.4 kg/m^2^ (range 18.9–42.5), and the mean eGFR was 75.5 ± 17.2 ml/min/1.73 m^2^ (range 6–90). All the other demographic and anthropometric data and laboratory values are presented in Table 1.

**Table 1:** Demographic, anthropometric and laboratory data of included patients (*n* = 133)

Parameter	Mean ± SD	Range
Age (years)	65.0 ± 9.2	27–82
Body Mass Index – BMI (kg/m^2^)	28.5 ± 4.4	18.9–42.5
Ankle-brachial index—ABI	1.0 ± 0.1	0.8–1.3
Creatinine (umol/L)	91.3 ± 65.4	49–666
Estimated glomerular filtration rate—eGFR (ml/min/1.73 m^2^)	75.5 ± 17.2	6–90
Cystatin C (mg/L)	1.1 ± 0.7	0.7–6.8
Hemoglobin (g/L)	139.8 ± 13.3	98–171
Cholesterol (mmol/L)	4.6 ± 1.1	2.0–7.3
Low-Density Lipoprotein cholesterol—LDL (mmol/L)	2.8 ± 1.0	0.9–6.0
High-Density Lipoprotein cholesterol—HDL (mmol/L)	1.3 ± 0.4	0.7–2.6
Triglycerides (mmol/L)	1.9 ± 1.1	0.4–5.8
N-terminal pro-hormone beta-natriuretic peptide – NT-pro-BNP (ng/L)	58.6 ± 185.7	3–2042

Arterial stiffness measurements are presented in Table 2.

**Table 2:** Pulse wave analysis and carotid-femoral pulse wave velocity measurements in included patients (*n* = 133)

Parameter	Mean ± SD	Range
Subendocardial viability ratio—SEVR (%)	164.4 ± 34.2	92–299
Augmentation index—AIx	29.1 ± 10.3	-8–56
AIx, normalized for heart rate 75/minute—AIx@75	26.3 ± 9.7	-13–59
Augmentation pressure—AP (mmHg)	15.0 ± 8.3	-3–47
Pulse pressure—PP (mmHg)	47.8 ± 14.5	17–94
Ejection duration—ED (ms)	33.8 ± 4.2	22–46
Aortic systolic pressure (mmHg)	127.2 ± 19.6	86–202
Aortic diastolic pressure (mmHg)	78.2 ± 13.1	42–110
Carotid-femoral pulse wave velocity—cfPWV (m/s)	10.3 ± 2.7	6.2–20.6

Coronarography showed normal coronary angiogram in 43 patients (32.3%), 24 patients had one-vessel coronary artery disease (18.0%), 28 patients had two-vessel coronary artery disease (21.1%) and 38 patients had three-vessel coronary artery disease (28.6%).

Comparing comorbidities, prescribed medications, BMI and arterial stiffness parameters between patients with different degrees of coronary artery disease showed no differences between groups. Comparison in arterial stiffness parameters in patients with different degrees of coronary artery disease is presented in Table 3.

**Table 3:** Arterial stiffness parameters in patients with different degrees of coronary artery disease (*n* = 133)

Arterial stiffness parameter	One-vessel coronary artery disease (*n* = 24)	Two-vessel coronary artery disease (*n* = 28)	Three-vessel coronary artery disease (*n* = 38)	No coronary artery disease (*n* = 43)	*p*
Subendocardial viability ratio – SEVR (%)	175.4 ± 36.2	163.6 ± 27.2	162.4 ± 38.3	160.5 ± 33.2	0.369
Carotid-femoral pulse wave velocity – cfPWV (m/s)	9.4 ± 2.0	10.6 ± 2.3	10.5 ± 2.7	10.5 ± 3.2	0.383
Augmentation index – AIx	30.0 ± 8.4	28.1 ± 8.5	29.1 ± 10.7	29.1 ± 12.1	0.940
Augmentation index, normalised for heart rate 75/minute (AIx@75)	25.6 ± 8.7	25.4 ± 7.3	26.2 ± 9.2	27.4 ± 12.0	0.824
Augmentation pressure – AP (mmHg)	13.3 ± 5.5	14.7 ± 6.9	16.4 ± 10.7	14.7 ± 7.9	0.538
Ejection duration – ED (ms)	32.5 ± 3.5	33.4 ± 4.0	34.1 ± 4.5	34.5 ± 4.4	0.272
Pulse pressure – PP (mmHg)	43.7 ± 13.5	48.5 ± 10.4	50.1 ± 17.5	47.7 ± 17.4	0.410
Central aortic systolic pressure (mmHg)	118.2 ± 14.6	125.7 ± 12.5	131.5 ± 26.9	129.3 ± 16.8	0.053
Central aortic diastolic pressure (mmHg)	74.6 ± 11.0	76.4 ± 10.5	78.4 ± 15.4	81.1 ± 13.1	0.227

BMI showed statistically significant correlation with SEVR (*r* = -0.193, *p* = 0.026), cfPWV (*r* = 0.417, *p* < 0.001) and PP (*r* = 0.227, *p* = 0.009). No correlation was found between BMI and ABI values or between BMI and eGFR. No correlation was found between BMI and coronary artery disease either.

Multiple regression analysis was performed to determine whether the correlation between BMI and several arterial stiffness parameters was independent of traditional atherosclerosis risk factors. Dependent variables were SEVR, cfPWV, and PP and independent variables were eGFR, age, diabetes mellitus, hyperlipidemia, BMI, and systolic blood pressure. A statistically significant and independent association was found between BMI and SEVR (B = -0.179, *p* = 0.044) and between BMI and cfPWV (B = 0.283, *p* < 0.001). Age also correlated with SEVR (B = -0.186, *p* = 0.037), cfPWV (B = 0.416, *p* < 0.001) and PP (B = 0.434, *p* < 0.001). No other associations were observed.

**Fig. 1:**
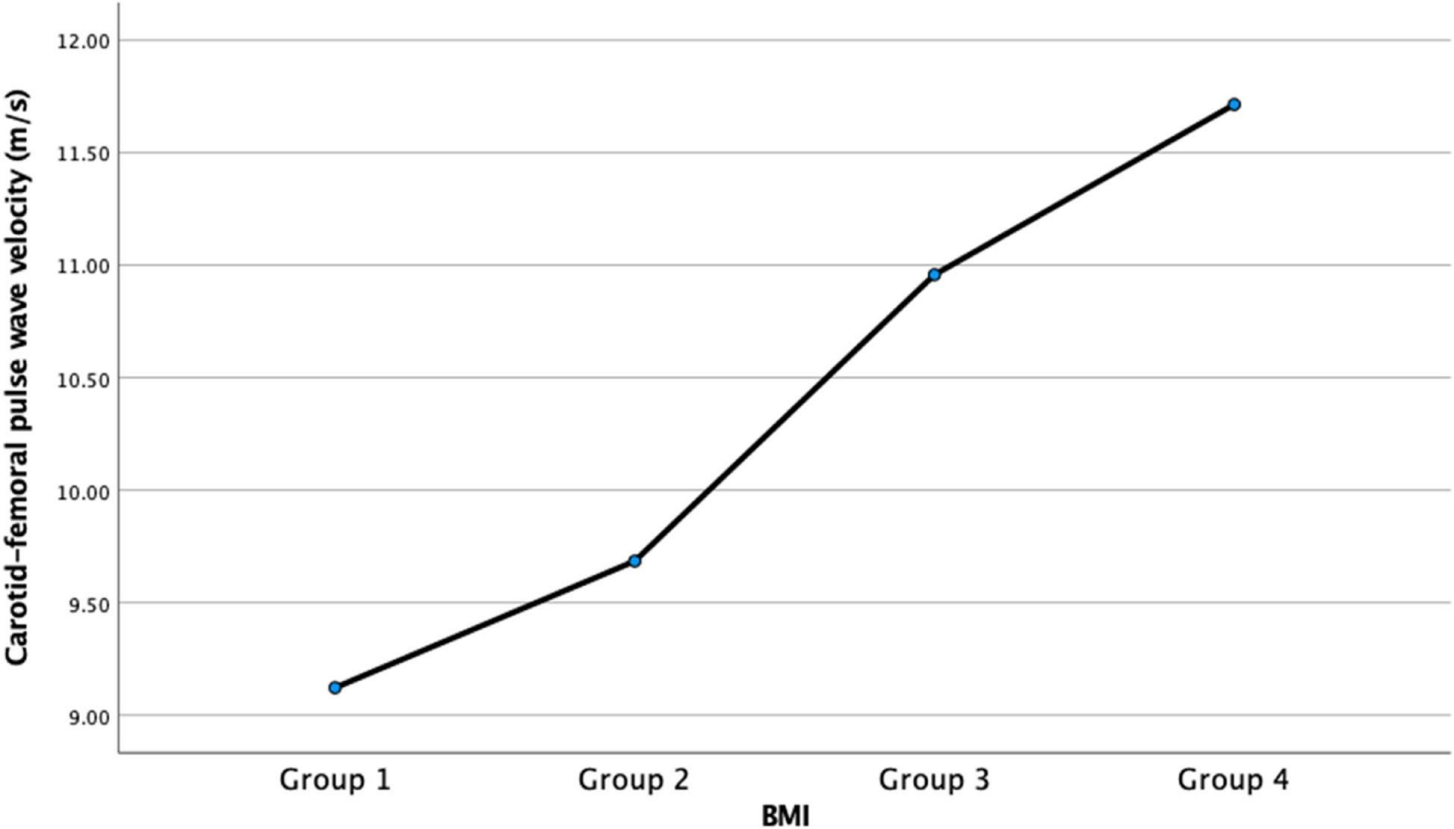
Carotid-femoral pulse wave velocities in different Body Mass Index (BMI) groups

**Fig. 2:**
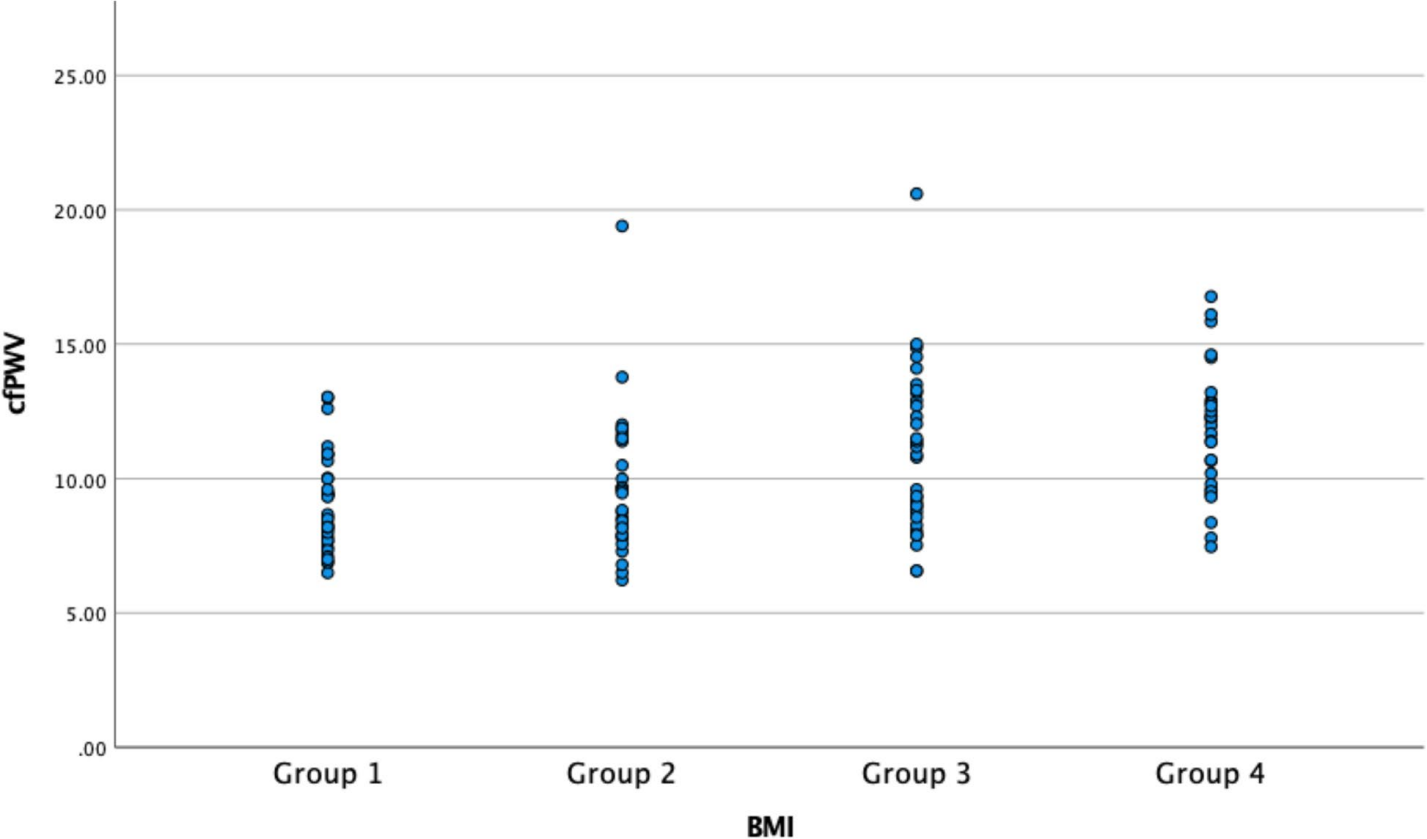
A scatterplot representing the differences in carotid-femoral pulse wave velocities between patients in different Body Mass Index (BMI) groups

Additionally, patients were divided into four groups, based on quartile values of BMI. In group 1, patients with BMI less than 25.2 kg/m^2^ were included, and group 2 included patients with BMI values between 25.2 and 28.5 kg/m^2^. In group 3, patients with BMI between 28.6 and 31.2 were included and patients with higher BMI values were placed in group 4. In Table 4, a comparative analysis of patients in different BMI groups is presented. The only statistically significant difference between the groups was in the values of cfPWV, which were progressively higher in patients with higher BMI values (Figs. 1 and 2). Patients in the different BMI groups did not differ in the degree of coronary artery disease (*p* = 0.232) (Table 5).

**Table 4:** The comparative analysis of patients in different BMI groups (*n* = 133)

Parameter	Group 1—BMI < 25.2 kg/m^2^ (*n* = 33)	Group 2—BMI 25.2 – 28.5 kg/m^2^ (*n* = 34)	Group 3—BMI 28.6 – 31.2 kg/m^2^ (*n* = 33)	Group 4—BMI > 31.2 kg/m^2^ (*n* = 33)	*p*
Age (years)	62.0 ± 10.5	64.7 ± 8.9	66.8 ± 9.2	66.1 ± 7.7	0.153
Creatinine (umol/L)	89.6 ± 74.4	86.1 ± 19.1	86.7 ± 31.6	85.3 ± 26.7	0.981
Estimated glomerular filtration rate – GFR (ml/min/1.73 m^2^)	79.9 ± 16.7	75.5 ± 15.3	74.2 ± 16.6	76.1 ± 16.2	0.515
Cystatin C (mg/L)	1.0 ± 0.8	1.0 ± 0.3	1.1 ± 0.5	1.1 ± 0.4	0.936
Mean ankle-brachial index—ABI	1.1 ± 0.1	1.0 ± 0.1	1.1 ± 0.1	1.1 ± 0.1	0.871
Subendocardial viability ratio – SEVR (%)	172.7 ± 44.0	171.2 ± 28.7	160.1 ± 31.2	154.8 ± 31.9	0.126
Carotid-femoral pulse wave velocity – cfPWV (m/s),	9.1 ± 1.8	9.7 ± 2.7	11.0 ± 3.0	11.7 ± 2.4	** < 0.001**
Pulse pressure – PP (mmHg)	47.7 ± 17.6	44.8 ± 12.0	47.5 ± 13.2	53.2 ± 13.6	0.131
Augmentation index – AIx	30.3 ± 11.2	29.8 ± 9.3	27.7 ± 9.4	29.3 ± 11.7	0.766
Augmentation pressure – AP (mmHg)	15.7 ± 10.0	14.7 ± 7.3	13.5 ± 6.2	17.0 ± 9.2	0.358
AIx, normalized for heart rate 75/minute – AIx@75	26.8 ± 13.3	26.0 ± 9.0	25.6 ± 8.4	27.0 ± 8.0	0.944
Ejection duration – ED (ms)	33.0 ± 4.5	32.9 ± 3.7	34.5 ± 4.3	34.5 ± 4.3	0.240
Aortic systolic pressure (mmHg)	127.4 ± 22.2	126.2 ± 20.6	127.7 ± 17.1	129.3 ± 19.8	0.944
Aortic diastolic pressure (mmHg)	78.3 ± 14.6	79.3 ± 10.0	79.8 ± 14.0	74.6 ± 13.7	0.391

**Table 5:** The comparison of the extent of coronary artery disease between patients in different BMI groups (*n* = 133). The difference was not statistically significant (*p* = 0.232)

Coronary artery disease	Group 1—BMI < 25.2 kg/m^2^ (*n* = 33)	Group 2—BMI 25.2 – 28.5 kg/m^2^ (*n* = 34)	Group 3—BMI 28.6 – 31.2 kg/m^2^ (*n* = 33)	Group 4—BMI > 31.2 kg/m^2^ (*n* = 33)
None	11 (33.3)	10 (29.4)	11 (33.3)	11 (33.3)
One-vessel (%)	7 (21.2)	6 (17.6)	5 (15.2)	6 (18.2)
Two-vessel (%)	6 (18.2)	7 (20.6)	8 (24.2)	7 (21.2)
Three-vessel (%)	9 (27.3)	11 (32.4)	9 (27.3)	9 (27.3)

## Discussion

The results of our study showed an important association between BMI and central arterial stiffness, independent of traditional atherosclerosis risk factors. No association was found between BMI, kidney function, ABI, and coronary artery disease. An obesity-driven, subclinical increase in arterial stiffness could therefore be the driving force antedating clinically detectable organ damage.

Patients in our study were mostly male and had high cardiovascular risk, based on their average age and several comorbidities. Most of the patients had normal ABI and eGFR values. Interestingly, despite previously positive non-invasive testing for myocardial ischemia, nearly one-third of patients had a normal coronary angiogram, without any signs of macrovascular coronary artery disease. After careful examination of their coronarographies, most of them had tortuous coronary arteries with slower blood flow, insinuating the presence of a non-obstructive form of coronary artery disease. Microvascular coronary artery disease is one of the forms of non-obstructive coronary artery disease, which can be present in up to 30% of patients with angina pectoris [23] and is associated with microvascular architectural changes and endothelial dysfunction [24]. According to some studies, decreased SEVR could be a marker of impaired coronary microcirculation and reduced coronary blood flow reserve in patients without significant macrovascular coronary pathology [25]. In our study, the differences in SEVR values between patients with different BMI values were not statistically significant, although patients with the highest BMI had slightly lower SEVR, especially if compared to patients in the lowest BMI group. Furthermore, the authors Pan et al. found that pulse wave velocity could be used as an effective indicator for the assessment of the microvascular biomechanical properties in rats [26]. Several studies have confirmed these findings in people, as well [27, 28]. Nonetheless, coronary blood flow reserve with intracoronary adenosine or acetylcholine application is still considered the best way to invasively test coronary microcirculation and endothelial function [29]. As this was not performed in our patients, the definitive diagnosis of microvascular coronary artery disease in this subgroup can be only presumptive and not definitive. Nonetheless, the possible finding of microvascular coronary disease is important, because it has potential therapeutic and prognostic implications, and can result in increased morbidity and mortality [30].

There have been some reports on the association between subendocardial perfusion and obesity. *Fantin *et al. performed a study on a cohort of 55 patients, half of them had clinical criteria for metabolic syndrome. Their findings suggested lower SEVR in patients with metabolic syndrome compared to the healthy cohort, even after the adjustment for age, sex, and mean arterial pressure. Their data included BMI, but they did not analyze their data for a potential correlation between BMI and SEVR [31]. In a study by *Strasser *et al., 146 participants were included. The authors found a statistically significant association between BMI and cfPWV [32]. Similarly, in a study by *Tocci *et al. which was performed on adolescent patients, the authors found overweight adolescents had higher cfPWV and systolic blood pressure and lower SEVR values, compared to normal-weight peers [33]. SEVR is a sensitive, easily reproducible, and validated measure of the balance between myocardial oxygen supply and demand, as well as of the adequacy of the subendocardial perfusion [34]. It is also an important marker of increased cardiovascular risk and is associated with increased cardiovascular mortality, especially in patients with CKD [35]. In a study by *Tsiachris *et al., the authors found a direct correlation between reduced coronary blood flow reserve (measured by intracoronary application of nitroglycerine) and reduced SEVR. This association was present even in the absence of any structural or functional left ventricle adaptations and also in the absence of macrovascular coronary artery disease. The authors concluded that SEVR was a reliable marker of microvascular dysfunction [25]. As evident from some animal models, obesity is independently interconnected with myocardial inflammation, oxidative stress, mitochondrial dysfunction, and myocardial fibrosis [36]. These findings could be extrapolated to our study. We believe the reduction in SEVR in patients with increased BMI was likely due to the direct impact of obesity on the structure of the myocardium and microvascular dysfunction, especially since neither traditional atherosclerosis risk factors nor macrovascular coronary artery disease correlated with BMI.

According to studies, increasing BMI is associated with decreasing arterial compliance, resulting in increased PP [37]. This was also our finding. Additionally, we found a graded association between BMI and cfPWV values, which were independent of traditional atherosclerosis risk factors. A study, which was performed on hypertensive patients with increased cardiovascular risk and was therefore methodologically similar to ours, found that increased BMI and cfPWV were strongly related and that body weight reduction was associated with an improvement in arterial distensibility and compliance [38]. Similarly, *Petersen *et al. found that even a modest decrease in body weight improved central arterial stiffness parameters [39, 40]. Similar findings were found in younger patients as well, emphasizing the impact of obesity on vessel adaptation and consequently reduced compliance [41]. The impact of obesity on arterial stiffness is multifactorial. Besides the conventional association with hyperlipidemia, arterial hypertension, diabetes, and CKD, obesity can lead to a state of an increased level of inflammation in the body, including vessel walls [42, 43]. Evidence shows increased material stiffness of the aortic intimal extracellular matrix, increased aortic wall inflammation, and endothelial permeability in obese individuals. These findings precede albuminuria, cardiac diastolic dysfunction, and hypertension [44]. Additionally, obesity might increase central arterial stiffness through leptin, which is a promotor of smooth muscle cell proliferation and angiogenesis [45]. It appears vascular inflammation and endothelial dysfunction could be key processes in obesity-related arterial stiffness changes observed in our patients, preceding clinically detectable target organ damage.

Our study had several limitations. The design of the study was imperfect in the sense that only patients with a high probability of cardiovascular disease were included. The study was not prospective and randomized, follow up measurements would be useful to assess the changes in arterial stiffness parameters during time in patients with different BMI. lso, only Caucasian patients were included, introducing racial bias into the results of the study. We did not perform definitive testing for microvascular coronary artery disease (scuh as coronary blood flow reserve with coronarography) and we also did not test for sensitive markers of inflammation, for example, high-sensitivity C-reactive protein. It would be sensible to perform additional research on a more diverse population and patients with different baseline cardiovascular risk profiles. Additionally, the inclusion of coronary blood flow reserve measurements, high-sensitivity C-reactive protein, and perhaps even echocardiography with strain measurements (to determine myocardial stiffening), would greatly improve our understanding of obesity-caused changes in vascular and myocardial function.

We believe that the results of our study, underlined with multifactorial analysis and the use of both non-invasive and invasive methods are important, and will hopefully inspire more researchers to perform additional studies on arterial stiffness changes in patients with increased BMI.

## Conclusion

Increased BMI is independently associated with increased central arterial stiffness, even in the absence of clinically detectable target organ damage.

## Data Availability

All data generated or analysed during this study are included in this published article.
